# Fibrodysplasic aneurysms of the extracranial internal carotid artery: a new case report

**DOI:** 10.11604/pamj.2017.28.170.5772

**Published:** 2017-10-23

**Authors:** Hamid Jiber, Youssef Zrihni, Hamza Naouli, Abdellatif Bouarhroum

**Affiliations:** 1 Department of Vascular Surgery, CHU Hassan II, Fes, Morocco

**Keywords:** Extracranial internal carotid artery aneurysm, fibromuscular dysplasia, surgical treatment

## Abstract

The other reports a case of fibrodysplasic aneurysm of the extracranial internal carotid artery that was successfully treated by resection and direct end-to-end anastomosis of the internal carotid artery.

## Introduction

Extracranial internal carotid artery aneurysms are rare, and according to their etiology they can be classified into atherosclerotic, dysplastic, inflammatory, infective or posttraumatic [1]. We describe a case of a true extracranial internal carotid artery aneurysm.

## Patient and observation

**Figure 1:**
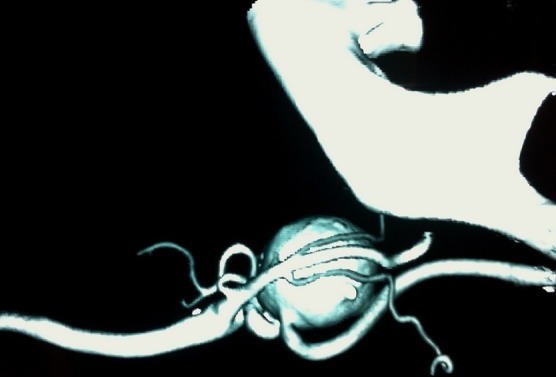
Reconstructed angio-CT scan showing a saccular aneurysm originating from the proximal left ICA

**Figure 2:**
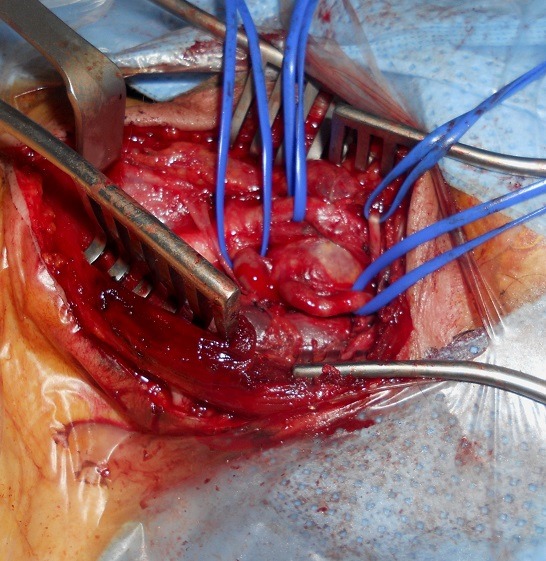
Intraoperative view: aneurysm at the origin of the right ICA

**Figure 3:**
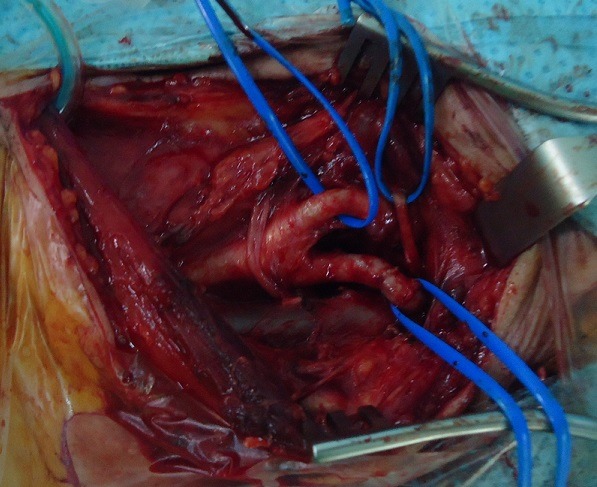
Intraoperative view: final result: end to end suture after total excision of the aneurysm

A 76-year-old woman with no history of cerebrovascular symptoms or cervical trauma was admitted to our hospital with a pulsatile left neck mass for five months before referral. Physical examination revealed a pulsatile solid cervical mass located anterior to the left sternocleidomastoid (SCM) muscle. There was no audible bruit over the carotid region. Diagnosis of extracranial internal carotid artery aneurysm was evocated. Duplex ultrasonography and-computed tomography scan (angio-CT scan) demonstrated a saccular aneurysm at the origin of the left Internal Carotid Artery (ICA) with a longitudinal diameter of 28mm and transversal diameters of 25,1mm x 25mm (Figure 1), the left carotid axis appeared free from calcific or stenotic plaque. In addition no obstructive or aneurysmal lesions of the contralateral carotid artery were detected. Laboratory tests were normal .The aneurysm was surgically treated under general anesthesia. The aneurysm was dissected and exposed by standard lateral cervical approach anterior to the SCM muscle (Figure 2). Simple proximal and distal arterial clamping after systemic administration of heparin (at 100 IU/kg body weight) was used during aneurysm repair. After aneurysmectomy end-to-end anastomosis of the ICA was performed because the distal carotid artery was significantly elongated and was of sufficient length (Figure 3). The time of carotid clamping was 25 minutes. No perioperative complications were encountered. Pathological study of the aneurysmatic carotid wall revealed fibromuscular dysplasia associated with thinning of the arterial wall, which was partially replaced with collagen fibers and proliferated smooth muscle cell bundles. The Patient was discharged from hospital on the fifth postoperative day without any neurological deficit. At one year follow-up the patient was asymptomatic without any neurological complication and with carotid patency.

## Discussion

Extracranial carotid artery (ECA) aneurysms are rare, only 0.1% to 2% of all surgical procedures of the ECA were performed for ECA aneurysms [2, 3]. The largest single center study was carried out by the Texas Heart Institute with 67 cases [2]. These lesions can be classified according to their different physiopathology into true or pseudoaneurysms. After excluding the cases of pseudoaneurysms secondary to the previous carotid endarterectomy or cervical trauma , true aneurysms are most commonly due to atherosclerosis or fibromuscolar dysplasia, although Moreou reported dysplastic lesions (single or associated with spontaneous dissection) as a major cause of ICA aneurysms [4].

The incidence of fibromuscular dysplasia was greatest in some reported series [5]. Whereas atherosclerosis was predominant in other series [2, 4, 6]. Faggioli et al. proposed that atherosclerotic changes may be a secondary process in the dysplastic artery [5]. Dysplastic aneurysms, often associated with chronic dissection are more distally located, whereas Atherosclerotic Aneurysms tend to be located in the common artery bifurcation and in the proximal ICA [7]. ECA aneurysms may be asymptomatic, but their natural history is associated with spontaneous progression of the aneurysm, most commonly associated with a high risk of neurological thromboembolic events, cranial nerve compression and, more rarely, rupture [3]. Although it is now the reference diagnostic method in the exploration of supra-aortic trunks, Doppler ultrasound may have limits in the identification and definition of extra-cranial carotid aneurysms. CT angiography proved to be an excellent tool to visualize the exact morphological details of the aneurysm [8]. Open surgery still remains the most valid option to prevent the most probable, severe and life-threatening complications, in particular embolisms [2].

In most saccular aneurysms, total resection of the aneurysm with direct end-to-end anastomosis of the ICA is feasible because the distal carotid artery is sufficiently elongated. When end-to-end anastomosis cannot be performed, interposition of the saphenous vein is advocated as the graft of choice [4, 9]. Prosthetic grafts have been also used; however, they are associated with a potential risk of late stenosis due to intimal hyperplasia [2]. Rarely performed, aneurysmorrhaphy followed by prosthetic patch angioplasty can be used in cases of saccular aneurysm [2, 4]. Controversies exist as to whether an intraluminal shunt should be used during aneurysm repair. Some surgeons use it routinely because of the prolonged clamp-occlusion time required for complicated repair of these aneurysms [10]. Some authors propose, however, that an intraluminal shunt is unnecessary if syncope or EEG changes do not occur during a 10-minute preoperative carotid artery occlusion test with a balloon catheter [11].

A review of the literature suggests that surgical repair has proven to be effective and safe [2], but endovascular treatment has been recently used in the treatment of ECA aneurysms by means of embolization with detachable coils, endografts or covered stents [12]. The use of covered stent allows simultaneous exclusion of the aneurysm and dilatation of an eventually stenosed distal ICA, with excellent results in terms of patency and absence of migration [13]. But studies of larger series of patients with extensive post-operative follow-up will be required to prove the safety and efficacy of endovascular procedures.

## Conclusion

Open surgery remains the gold standard for the treatment of extracranial internal carotid artery aneurysms in terms of patency and reduced risk of adverse complications, end-to-end anastomosis of the internal carotid artery is the preferred method of repair if the length of the distal internal carotid artery permits; endovascular procedures may, in selected cases, may be considered as a therapeutic choice.

## Competing interests

Authors declare no competing interests.
